# Data of enzymatic activities of the electron transport chain and ATP synthase complexes in mouse hepatoma cells following exposure to 2,3,7,8-tetrachlorodibenzo-p-dioxin (TCDD)

**DOI:** 10.1016/j.dib.2016.05.018

**Published:** 2016-05-20

**Authors:** Hye Jin Hwang, Michelle Steidemann, Taylor K. Dunivin, Mike Rizzo, John J. LaPres

**Affiliations:** a Department of Biochemistry and Molecular Biology, Michigan State University, East Lansing, MI 48824, United States; b Center for Mitochondrial Science and Medicine, Michigan State University, East Lansing, MI 48824, United States; c Institute for Integrative Toxicology, Michigan State University, East Lansing, MI 48824-1319, United States; d Department of Pharmacology and Toxicology, Michigan State University, East Lansing, MI 48824, United States; e Department of Microbiology and Molecular Genetics, Michigan State University, East Lansing, MI 48824, United States; f Cell and Molecular Biology Graduate Program, Michigan State University, East Lansing, MI 48824, United States

**Keywords:** AHR, Aryl hydrocarbon receptor, TCDD, Electron transport chain, Mitochondria

## Abstract

2,3,7,8-tetrachlorodibenzo-p-dioxin (TCDD) is the most widely studied ligand of the aryl hydrocarbon receptor (AHR). The AHR-dependent TCDD-induced mitochondrial hyperpolarization (Tappenden et al., 2011) [1] and reduced oxygen consumption rate of intact mouse hepatoma cells (Huang et al., in press) [2] in the previous studies suggest that these alterations can be related to enzymatic activities of the electron transport chain (ETC) and ATP synthase in oxidative phosphorylation (OXPHOS) system. Here, we evaluated the activity of each complex in the OXPHOS system using *in vitro* enzymatic assays. The calculated enzymatic activity of each complex was normalized against the activity of citrate synthase. To combine each value from an independent experiment, normalized enzyme activities from cells exposed to TCDD were converted to fold changes via comparison to the activity relative to time-matched vehicle control. The averaged fold change for each treatment suggests more replicates are needed in order to clearly evaluate a difference between treatments.

**Specifications Table**

**Table:** 

Subject area	*Biochemistry, Toxicology*
More specific subject area	*Enzyme kinetics*
Type of data	*Graph*
How data was acquired	*Spectrometry (*SpectraMax M2 spectrophotometer, Molecular Devices,);
Data format	*Analyzed*
Experimental factors	*Hepa1c1c7 and Hepa-C12 cells were exposed to 30* *nM 2,3,7,8-tetrachlorodibenzo-p-dioxin (TCDD) or* 0.01% DMSO *for 6 and 24 h.*
Experimental features	*Mitochondria were isolated from each cell line following DMSO/TCDD treatment and used for each enzymatic assay of the electron transport chain (ETC) and ATP synthase.*
Data source location	*East Lansing, MI, USA*
Data accessibility	*Data are within this article*

## **Value of the data**

• These *in vitro* data can be used to help explain other TCDD-induced changes in mitochondrial function.
• These data can help explain the direct impact of TCDD and the AHR on the respiratory chain.
• This data can help understand the role of mitochondria in AHR-dependent TCDD-induced toxicity.

## 1 Data

**Fig. 1:**
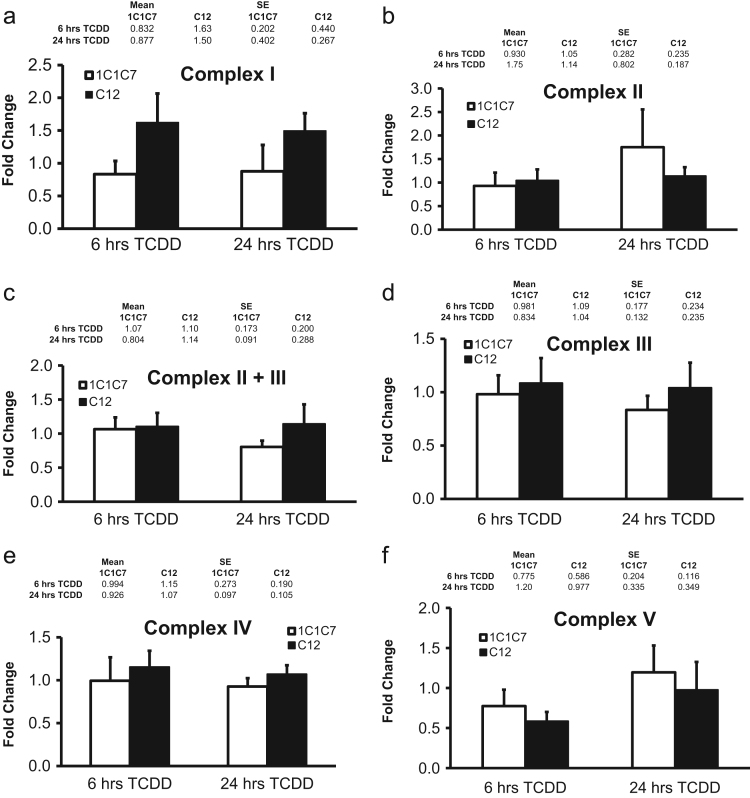
Activities of ETC complexes and ATP synthase.The activities of the ETC complexes and ATP synthase were measured with isolated mitochondria from hepa1c1c7 and c12 cells exposed to 30 nM TCDD or vehicle control (DMSO 0.01%) for 6 and 24 h. The activity of each complex (A. Complex I, B. Complex II, C. Complex II+III, D. Complex III, E. Complex IV, and F. Complex V) was normalized against the activity of citrate synthase. The fold change was calculated by re-normalization of enzyme activity from cells exposed to TCDD with the activity relative to time-matched vehicle control. The bars represent mean±the standard errors (*n*=4).

We evaluated AHR-dependent changes in enzyme activity of the OXPHOS system in mouse hepatoma cells following exposure to TCDD (30 nM) for 6 and 24 h. Each enzymatic activity was normalized against citrate synthase activity, which represents an assessment of mitochondrial amount and integrity for each sample. Data is presented as a fold change by re-normalizing citrate synthase normalized enzymatic activities from cells exposed to TCDD against the activity relative to a time-matched vehicle (DMSO) control (Fig. 1). The variability of this data suggests more replicates than are obtained here (*n*=4) are needed to clearly understand AHR-dependent TCDD-induced mitochondrial dysfunction.

## 2 Experimental design, materials and methods

### 2.1 Cell culture

The mouse hepatoma cell line, hepa1c1c7 (AHR-expressing) and hepac12 (AHR-deficient), were grown as described previously [1], [2].

### 2.2 TCDD exposure

Hepa1c1c7 and c12 cells were exposed to 30 nM TCDD or 0.01% DMSO as a vehicle control for 6 or 24 h when cells had reached 70% confluency.

### 2.3 Preparation of mitochondrial proteins

TCDD or DMSO-treated cells were harvested and mitochondrial fractions were isolated using protocols as described previously [1], [2].

### 2.4 Enzymatic assay of OXPHOS system

Each enzyme solution was prepared by suspension of mitochondrial pellets either in a hypotonic buffer [25 mM potassium phosphate (KPO_4_) (pH 7.4) and 5 mM MgCl_2_] for citrate synthase, complex I, complex II, complex IV, and complex V or in mitochondrial buffer for complex II+III and complex III. After three cycles of freezing/thawing, protein concentrations were determined using Bio-Rad Bradford assay kit (Hercules, CA). Typical protein concentrations were 1.5~2 μg/μL. The activities of the individual ETC complexes, ATP synthase and citrate synthase were determined as previously described with slight modifications [3], [4]. The enzyme activities were determined at 37 °C for complex I and V and at 30 °C for the other ETC complexes and citrate synthase. The enzyme activity was calculated by

(ΔAbs/min)×(total assay volume)/[*ε*×(mitochondrial volume)×(mitochondrial concentration)]

with units of micromoles per min per milligram (*ε*: extinction coefficient). The enzymatic activity was calculated as a ratio, dividing each activity in micromoles per min per milligram protein by the citrate synthase activity. The absorbance for each enzymatic activity was measured using a SpectraMax M2 spectrophotometer (Molecular Devices, Sunnyvale, CA). In all cases, cytochrome c was bovine heart cytochrome c.

#### 2.4.1 Citrate synthase assay

5 μg of each enzyme solution was incubated with citrate synthase buffer [50 mM KPO_4_ (pH 7.4), and 0.1 mM 5,5′-Dithiobis(2-nitrobenzoic acid)] containing 100 μM acetyl CoA, in a cuvette for 5 min. The change of absorbance was measured at 412 nm for 2 min for reference. After addition of 100 μM oxaloacetate, the change in absorbance at 412 nm was recorded for 3 min. Enzyme activity was calculated with *ε* for the thionitrobenzoate anion (13.6 mM^−1^ cm^−1^).

#### 2.4.2 Complex I assay

50 μg of each enzyme solution was mixed with complex I buffer [50 mM KPO_4_ (pH 7.4), 140 μM β-Nicotinamide adenine dinucleotide (NADH), 1 mM potassium cyanide (KCN), 10 μM antimycin A, 0.1% BSA, and 50 μM 2,6-dichlorophenolindophenol (DCPIP)] with 1% ethanol and 50 μM Coenzyme Q_1_ (CoQ_1_) in a cuvette. The change in absorbance at 340 nm was recorded for 3 min. Reference was measured in the presence of 2.5 μM rotenone (dissolved in ethanol). Enzyme activity was calculated with *ε* for the NADH (6.22 mM^−1^ cm^−1^).

#### 2.4.3 Complex II assay

10 μg of each enzyme solution was incubated with complex II buffer [50 mM KPO_4_ (pH 7.4), 10 mM succinate, 1 mM KCN, 2.5 μM rotenone, and 10 μM antimycin A] for 10 min in a cuvette. After addition of 50 μM DCPIP, the change in absorbance at 600 nm was recorded for 2 min for reference. The change in absorbance at 600 nm was then recorded for 3 min in the presence of 50 μM CoQ_1_. Enzyme activity was calculated with *ε* for the DCPIP (19.1 mM^−1^ cm^−1^).

#### 2.4.4 Complex II+III assay

10 μg of each enzyme solution was incubated with complex II+III buffer [50 mM KPO_4_ (pH 7.4), 10 mM succinate, 1 mM KCN, 2.5 μM rotenone, 0.1% BSA, 0.075% EDTA, and 1 mM ATP] in a cuvette for 5 min. Upon addition of 32 μM cytochrome c, absorbance at 550 nm was recorded for 5 min. The reference was measured in the absence of cytochrome c. Enzyme activity was calculated with *ε* for the reduced cytochrome c (19.6 mM^−1^ cm^−1^).

#### 2.4.5 Complex III assay

5 μg of each enzyme solution was mixed with complex III buffer [50 mM KPO_4_ (pH 7.4), 1 mM n-dodecyl maltoside, 1 mM KCN, 2.5 μM rotenone, and 0.1% BSA] with 100 μM decylbenzolquinol and 30 μM cytochrome c in a cuvette. The change in absorbance at 550 nm was recorded for 3 min. Cytochrome c was fully reduced at the end of the measurement with dithionite. The reference was measured without enzyme solution. Enzyme activity was calculated with *ε* for the reduced cytochrome c (19.6 mM^−1^cm^−1^).

#### 2.4.6 Complex IV assay

Reduced cytochrome c was prepared using sodium dithionite. 10 μg of each enzyme solution was mixed with complex IV buffer [40 mM KPO_4_ (pH 6.8), 0.5% Tween 80, and 0.4 mg/mL reduced cytochrome c] in a cuvette. The change in absorbance at 550 nm was recorded for 2 min. 5 mM potassium ferricyanide was added to fully oxidized cytochrome c at the end of the measurement. The reference was measured without enzyme solution. Enzyme activity was calculated with *ε* for the reduced cytochrome c (19.6 mM^−1^ cm^−1^).

#### 2.4.7 Complex V assay

Complex V buffer [40 mM Tris-HCO_3_ (pH 8.0), 1 mM EGTA, 5 mM MgCl_2_, 0.2 mM NADH, 2.5 mM phosphoenolpyruvate, 0.5 μM antimycin A, 15 μM Carbonyl cyanide 3-chlorophenylhydrazone, 50 μg/mL lactate dehydrogenase, and 50 μg/mL pyruvate kinase] was incubated with 2.5 mM ATP for 2 min in a cuvette. After 10 μg of each enzyme solution was added to the above mixture, the change in absorbance at 340 nm was recorded for 5 min. The reference was measured in the presence of 2 μM oligomycin for 5 min. Enzyme activity was calculated with *ε* for the NADH (6.22 mM^−1^ cm^−1^).
